# *Phosphate transporter* gene families in rye (*Secale cereale* L.) – genome-wide identification, characterization and sequence diversity assessment via DArTreseq

**DOI:** 10.3389/fpls.2025.1529358

**Published:** 2025-06-16

**Authors:** David Chan-Rodriguez, Brian Wakimwayi Koboyi, Sirine Werghi, Bradley J. Till, Julia Maksymiuk, Fatemeh Shoormij, Abuya Hilderlith, Anna Hawliczek, Maksymilian Królik, Hanna Bolibok-Brągoszewska

**Affiliations:** ^1^ Department of Plant Genetics Breeding and Biotechnology, Institute of Biology, Warsaw University of Life Sciences, Warsaw, Poland; ^2^ Veterinary Genetics Laboratory, University of California, Davis, Davis, CA, United States; ^3^ Department of Agronomy and Plant Breeding, College of Agriculture, Isfahan University of Technology, Isfahan, Iran

**Keywords:** rye, *Secale cereale* L., *Pht* genes, phosphate deficiency, phylogenetic relationships, gene diversity, low-coverage resequencing, expression profiling

## Abstract

Phosphorus is a macronutrient indispensable for plant growth and development. Plants utilize specialized transporters (PHT) to take up inorganic phosphorus and distribute it throughout the plant. The PHT transporters are divided into five families: PHT1 to PHT5. Each PHT family has a particular physiological and cellular function. Rye (*Secale cereale* L.) is a member of *Triticeae*, and an important source of variation for wheat breeding. It is considered to have the highest tolerance of nutrient deficiency, among *Triticeae*. To date, there is no report about genes involved in response to phosphorus deficiency in rye. The aim of this study was to: (i) identify and characterize putative members of different phosphate transporter families in rye, (ii) assess their sequence diversity in a collection of 94 diverse rye accessions via low-coverage resequencing (DArTreseq), and (iii) evaluate the expression of putative rye *Pht* genes under phosphate-deficient conditions. We identified 29 and 35 putative *Pht* transporter genes in the rye Lo7 and Weining reference genomes, respectively, representing all known *Pht* families. Phylogenetic analysis revealed a close relationship of rye PHT with previously characterized PHT proteins from other species. Quantitative RT PCR carried out on leaf and root samples of Lo7 plants grown in Pi-deficient and control condition demonstrated that *ScPht1;6*, *ScPht2* and *ScPht3;3* are Pi-deficiency responsive. Based on DArTreseq genotyping of 94 diverse rye accessions we identified 820 polymorphic sites within rye *ScPht*, including 12 variants identified by the SIFT algorithm as having a potentially deleterious effect, of which three are scored as high confidence. SNP density varied markedly between *ScPht* genes. This report is the first step toward elucidating the mechanisms of rye’s response to Pi deficiency. Our findings point to multiple layers of adaptation to local environments, ranging from gene copy number variation to differences in level of polymorphism across *Pht* family members. DArTreseq genotyping permits for a quick and cost-effective assessment of polymorphism levels across genes/gene families and supports identification and prioritization of candidates for further studies. Collectively our findings provide the foundation for selecting most promising candidates for further functional characterization.

## Introduction

Plants are sedentary organisms, forced to rely on the supply of nutrients available in their immediate vicinity. Phosphorus (P) is a macronutrient indispensable for plant growth and development, especially for the development of roots. At the cellular level, various metabolic processes, such as synthesis of nucleic acids, require P (Chithrameenal et al., 2018). During evolution, plants have developed complex and intricate mechanisms of efficient nutrient uptake and utilization. During low P conditions, the expression of genes involved in orthophosphate (Pi) mobilization increases (Młodzińska and Zboińska, 2016). These genes collectively are known as phosphate-starvation inducible genes (PSI). The molecular mechanism governing the transcriptional regulation of PSI genes – which include *phosphate transporters* – has been extensively studied in *Arabidopsis thaliana*. For instance, the transcriptional factor Phosphate starvation response 1 (PHR1) binds the *cis*-regulatory element P1BS (PHR1-binding site) to positively regulate the expression of PSI, which includes some members of the *Pht* gene families (Puga et al., 2014). Similarly, Pi deficiency regulates the rice (*Oryza sativa*) PSI genes, suggesting a conserved regulatory mechanism across plant species (Wang et al., 2014b).

Plants utilize specialized transporters to take up inorganic Pi from the rhizosphere and distribute it throughout the plant. The PHT transporters are divided into five families, namely PHT1, PHT2, PHT3, PHT4, and PHT5. Each PHT transporter family has a particular physiological and cellular function in plants (Liu et al., 2016b; Zhang et al., 2016). The PHT transporter family sizes vary across the plant species. For example, Arabidopsis and rice differ in the number of PHT1 and PHT3 members. The Arabidopsis PHT1 family includes nine members, while the rice PHT1 family contains 13. The PHT3 family has three and six members in Arabidopsis and rice, respectively (Młodzińska and Zboińska, 2016). The members of the membrane-localized PHOSPHATE TRANSPORTER 1 (PHT1) family are associated with the primary uptake, translocation, and allocation of Pi (Wang et al., 2017, 2021). Some PHT1 members are strongly expressed in vegetative (leaves, stems, and roots) and reproductive tissues (flowers) under Pi deficiency (Nussaume et al., 2011). The other transporter families mobilize Pi to internal compartments such as the chloroplast (PHT2), mitochondria (PHT3), Golgi and plastids (PHT4), and vacuole (PHT5) (Wang et al., 2017, 2021). The PHT1 transporter family has been extensively studied in crop plants including rice (Liu et al., 2011), barley (Preuss et al., 2010), maize (Liu et al., 2016a), wheat (Teng et al., 2017), *Setaria italica* (Ceasar et al., 2014), sorghum (Walder et al., 2015; Maharajan et al., 2021), finger millet (Pudake et al., 2017) and poplar (Zhang et al., 2016). PHT2 mobilize Pi to chloroplast. Unlike the rest of the Pi transporters, the PHT2 family contains a single member in most plants and their expression is restricted to leaves (Rausch et al., 2004). PHT3 proteins are located in the mitochondria and mediate the distribution of Pi between the inner mitochondrial membrane and cytoplasm (Rausch and Bucher, 2002; Zhu et al., 2012). PHT4 proteins are localized in the chloroplasts, the Golgi apparatus and non-photosynthetic plastids (Guo et al., 2008). In Arabidopsis, the PHT5 imports Pi to the vacuole to maintain the Pi homeostasis in the cytoplasm (Liu et al., 2016b).

The natural genetic variation within plant species is crucial for identifying unique alleles or rare genetic variants to develop more resilient and productive plant varieties. Various efforts have been made to map loci associated with low P tolerance in model plants and crops. Natural allelic variations associated with P deficiency tolerance were identified so far in several plant species (mostly via genome-wide association studies - GWAS), such *Aegilops tauschii* (Liu et al., 2015), Arabidopsis (Bouain et al., 2016, 2019), soybean (Ning et al., 2016), wheat (Lin et al., 2020; Soumya et al., 2021), maize (Luo et al., 2024), and rice (Yan et al., 2023). The *PSTOL1* gene is an example of the potential of natural variation in crop improvement for P deficiency tolerance in rice. This gene is absent from the P-starvation-sensitive rice variety Nipponbare, but present in the P-deficiency-tolerant variety Kasalath – a traditional rice variety (Gamuyao et al., 2012).

Rye (*Secale cereale* L.) is a cereal especially popular in Central, Eastern and Northern Europe. Poland is the world’s second largest rye grain producer (http://www.fao.org/faostat). Genetically rye is a diploid, with a very large (8Gpz) and highly repetitive genome (Rabanus-Wallace et al., 2021). Rye is a member of *Triticeae*, closely related to wheat and barley, and an important source of variation for wheat breeding (Crespo-Herrera et al., 2017). Frequently grown on marginal soils, rye is considered to have the highest tolerance of abiotic stresses, such as frost and drought, among *Triticeae*, and the highest tolerance of nutrient deficiency (Geiger and Miedaner, 2009; Rakoczy-Trojanowska et al., 2021). Contrary to barley and wheat, rye is out-crossing, and has a genetic self-incompatibility mechanism. Therefore, the development of homozygous lines for breeding and research is a challenging task. The rye genetic diversity remains underutilized as a nutrient-deficiency-tolerant allele resource for crop improvement, and there is a lack of knowledge about the genetic basis of P deficiency tolerance in rye.

This study identified and characterized putative members of different phosphate transporter families in *Secale cereale* L. Lo7 (Rabanus-Wallace et al., 2021) and Weining reference (Li et al., 2021) genomes. Our study also assessed the sequence diversity of the identified putative rye phosphate transporters (*ScPht*) in a collection of 94 diverse rye accessions via low-coverage resequencing (DArTreseq). Furthermore, we evaluated the gene expression of some of these putative *ScPht* under Pi-deficient conditions.

## Methods

### Plant material and growth conditions

Rye inbred line Lo7 was grown hydroponically under Pi-deficient (0.004 mM KH_2_PO_4_) and control (0.2 mM KH_2_PO_4_) conditions, using a nutrient solution based on (Yuan et al., 2017). Seeds were germinated on moist horticultural pumice gravel. After six days, uniformly germinated seedlings without residual endosperm were transferred to hydroponics. Plants were grown in a growth chamber at 19°C and photon fluency rate of ca. – 500 µmol m^-2^s^-1^, (14 hours day/10 hours night). Nutrient solution was exchanged three times per week. Leaf and root samples were collected on days 14 and 21 of the hydroponic trial, frozen in liquid nitrogen and stored at −80°C.

DArTreseq genotyping was carried out on DNA samples of 94 rye accessions representing a broad spectrum of rye genetic variation in terms of geographic origin and improvement status. Detailed information on the accessions used in this study is provided in the **Supplementary Table S1**. Each accession was represented by a single plant. Tissue collection, as well as DNA isolation using Mag-Bind Plant DNA DS Kit (OMEGA Bio-Tek) was carried out as described in (Hawliczek et al., 2020).

### Identification of *Pht* genes in the Lo7 and Weining rye genomes

Putative phosphate transporters belonging to the PHT1, PHT2, PHT3, PHT4 and PHT5 families were identified in the inbred line Lo7 and the Chinese elite rye Weining genomes using both the TBLASTN algorithm within the IPK Galaxy Blast Suite (https://galaxy-web.ipk-gatersleben.de/) and in WheatOmics 1.0 web platform (http://wheatomics.sdau.edu.cn), respectively. We used the Arabidopsis and rice PHT transporters - PHT1.1 (NP_199149.1; AAN39042.1), PHT2.1 (NP_189289.2; XP_015626495.1), PHT3.1 (NP_196908.1; NP_001406294.1), PHT4.1 (NP_180526.1; BAS71585.1) and PHT5.1 (NP_001185297.1) - as protein sequence queries. The rye Lo7 and Weining *Pht* coding sequences were retrieved from the IPK Galaxy Blast Suite and WheatOmics, respectively. The chromosome diagrams showing the location of the *Pht* genes were generated using the Phenogram web tool (https://visualization.ritchielab.org/phenograms/plot). The figure indicating putative orthologs synteny and gene duplications in Lo7 and Weining genomes was drawn using Circos (Krzywinski et al., 2009).

### Synteny and collinearity analysis

The rye genomes (Lo7 and Weining) sequence and annotation files were obtained from Ensemble plants (https://plants.ensembl.org) and the Chinese National Genomic Center (https://ngdc.cncb.ac.cn/gwh/Assembly/12832/show). We detected gene syntenic blocks between Lo7 and Weining genomes using MCScanX within the TBtools software (Wang et al., 2012; Chen et al., 2020). The MCScanX collinearity results were visualized using SynVisions (Bandi and Gutwin, 2020).

### Gene structure and amino acid sequence analysis

We obtained information about the exon-intron structure of each putative rye *Pht* from the Ensembl plant database (https://plants.ensembl.org/Secale_cereale/Info/Index). The predicted amino acid sequences for all putative rye PHT were retrieved using the ORFinder tool from NCBI (https://www.ncbi.nlm.nih.gov/orffinder/) and the Ensembl plants database. We examined the protein sequences for conserved domains using the InterPro (https://www.ebi.ac.uk/interpro/) and SMART (http://smart.embl.de/) tools. We predicted the subcellular localization and the transmembrane helixes for each rye PHT member using the Plant-mPLoc database (http://www.csbio.sjtu.edu.cn/bioinf/plant-multi/), Plant-mSubP (http://bioinfo.usu.edu/Plant-mSubP/) and TMHMM Server v. 2.0 web tool (https://services.healthtech.dtu.dk/services/TMHMM-2.0/). The chromosome diagrams showing the location of the *Pht* genes were generated using the Phenogram web tool (https://visualization.ritchielab.org/phenograms/plot). The figure indicating putative orthologs synteny and gene duplications in Lo7 and Weining genomes was drawn using Circos (Krzywinski et al., 2009). We generated the gene structure and protein conserved domain diagram using the Gene Structure Display Server (GSDS 2.0) and TBtools, respectively (Hu et al., 2015; Chen et al., 2020).

### Analysis of the *ScPht*s promoter regions

We analyzed the 2 Kb upstream region of the rye *Pht* genes using the plant *cis*-acting regulatory DNA element database PLACE (www.dna.affrc.go.jp/PLACE/?action=newplace). The upstream region sequences for all putative rye Lo7 *Pht* genes were obtained from the IPK Galaxy Blast Suite (https://galaxy-web.ipk-gatersleben.de/) and the Ensembl plants database (https://plants.ensembl.org/Secale_cereale/Info/Index).

### Phylogenetic analysis

We performed multiple alignments of the PHT protein sequences from *Secale cereale* Lo7 and Weining, *Oryza sativa*, *Sorghum bicolor*, *Glycine max* and *Arabidopsis thaliana* using Clustal W in MEGA X (Kumar et al., 2018). The Arabidopsis, rice, soybean, and sorghum PHTs protein sequences were retrieved from the Phytozome database (https://phytozome-next.jgi.doe.gov) (**Supplementary Table S2**). The unrooted PHT protein tree, was generated using the maximum likelihood method implemented in the IQTREE web tool (Trifinopoulos et al., 2016) with ultrafast bootstrapping (1000 replicates) and single branch test (SH-aLRT 1000 replicates). We used ModelFinder to select the WAG+G4+F model as the best-fitting substitution model for our PHT dataset (Kalyaanamoorthy et al., 2017). For the phylogenetic analysis of monocot (grasses) PHT1 transporters, we carried out multiple alignments of PHT1 protein sequences of *Secale cereale*, *Triticum aestivum*, *Hordeum vulgare*, *Oryza sativa*, *Sorghum bicolor*, *Zea mays*, *Setaria viridis*, *Brachypodium distachyon*, *Glycine max*, and *Arabidopsis thaliana*. The PHT1 protein tree was built following the parameters described above using the JTT+R5 as the best-fitting substitution model as determined by ModelFinder. We used Figtree v1.4.4 software (https://github.com/rambaut/figtree) to further annotate and visualize the protein phylogenetic tree.

### Quantitative RT-PCR

Total RNA was extracted using the Universal RNA Purification Kit (EURX, Gdansk, Poland) according to the manufacturer’s instructions. RNA was assessed for quality using a Nabi UV/Vis Nano Spectrophotometer and for integrity on 2% denaturing agarose gel. cDNA was synthesized from ~1 μg RNA using the RevertAid First Strand cDNA Synthesis Kit (Thermo Fisher Scientific, Lithuania). Quantitative PCR was performed in a 20 μl reaction containing: 10 μl of FastStart Essential DNA Green Master (Roche Diagnostics, Mannheim, Germany), 8μl of 2.5 ng µl-1 cDNA, and 1 μl of 10 mM of each primer using LightCycler^®^ 96 System (Roche Diagnostics, Mannheim, Germany). The cycling conditions were: one cycle at 95°C for 10’, followed by 40 cycles at 95°C for 10 s, 60°C for 10 s, and 72°C for 15 s, and the melting step - 95°C for 10 s, 65°C for 60 s and 97°C for 1 s. Reference genes *ScActine* and *ScEFa1* were used (Zhan et al., 2023). The coding sequences of *ScPht* genes were aligned using the Clustal Omega web tool (Madeira et al., 2024). Multiple primers specific to the genes: *ScPht1;6*, *ScPht1;7*, *ScPht1;11*, *ScPht2*, *ScPht3;1*, *ScPht3;3, ScPht3;4* and *ScPht5;3* were designed using Primer-BLAST (Ye et al., 2012). Only primers that demonstrated an efficiency of at least 90% were used for the experiment. RT-qPCR experiments and analysis were done according to MIQE guidelines (Bustin et al., 2009). Analyses were performed in two technical and three biological replicates. Reactions for the reference gene were included in each plate. Data from RT-qPCR were analyzed using the 2–ΔΔCt method (Livak and Schmittgen, 2001). Statistical significance was assessed with the Kruskal test, and plots were generated in R (R_Core_Team, 2013) using packages ggplot2 (v3.3.3) (Wickham, 2016) and dplyr (v0.7.6) (https://github.com/tidyverse/dplyr).

### DArTreseq^®^ genotyping - evaluation of sequence diversity of putative *ScPht* genes

Genotyping via DArTreseq^®^ was carried out at Diversity Arrays (Bruce, ACT, Australia). This approach, involving an effective depletion of repetitive sequences, represents a novel way of acquiring a very detailed genome profile in a more cost-effective manner than the Whole Genome Sequencing (WGS) methods. For removal of methylated genomic DNA each sample was digested with *MspJI* (New England Biolabs, Massachusetts, USA). After purification with AMPure beads (Beckman Coulter, California, USA) DNA fragments larger than 300 bp were retained and used for library preparation with Nextera DNA Flex Library Prep kit (Illumina, California, USA). Sequencing was performed on a 150 cycles pair-end run on Illumina Novaseq6000 sequencer (California, USA) to achieve 10 to 20 million reads per sample. Raw sequence reads were processed in DArTreseq pipeline. Poor quality reads were discarded and remaining sequences aligned to Lo7 genome sequence (Rabanus-Wallace et al., 2021). FreeBayes (https://github.com/freebayes/freebayes) was used to call SNP markers. Genetic variants were annotated for their predicted effect on gene function using SIFT (Vaser et al., 2016). A SIFT database was created using the Lo7 genome reference, the associated GFF3 annotation file converted to GTF using gffread, and the UniRef90 protein database using SIFT4G_Create_Genomic_DB (Pertea and Pertea, 2020). The effect of missense changes on protein function were predicted using the SIFT4G annotator tool (Vaser et al., 2016). For evaluation of genetic diversity of 94 rye accessions SNPs with MAF > 0.01 and <10% missing data were identified. Then, an Euclidean distance matrix was calculated using R package ‘stats’ (R_Core_Team, 2013) and used for NJ tree construction in MEGA11 (Tamura et al., 2021) and principal coordinates analysis in GenAlEx 6.5 (Peakall and Smouse, 2006, 2012).

## Results

### Identification of *Pht* family members in rye

**Figure 1:**
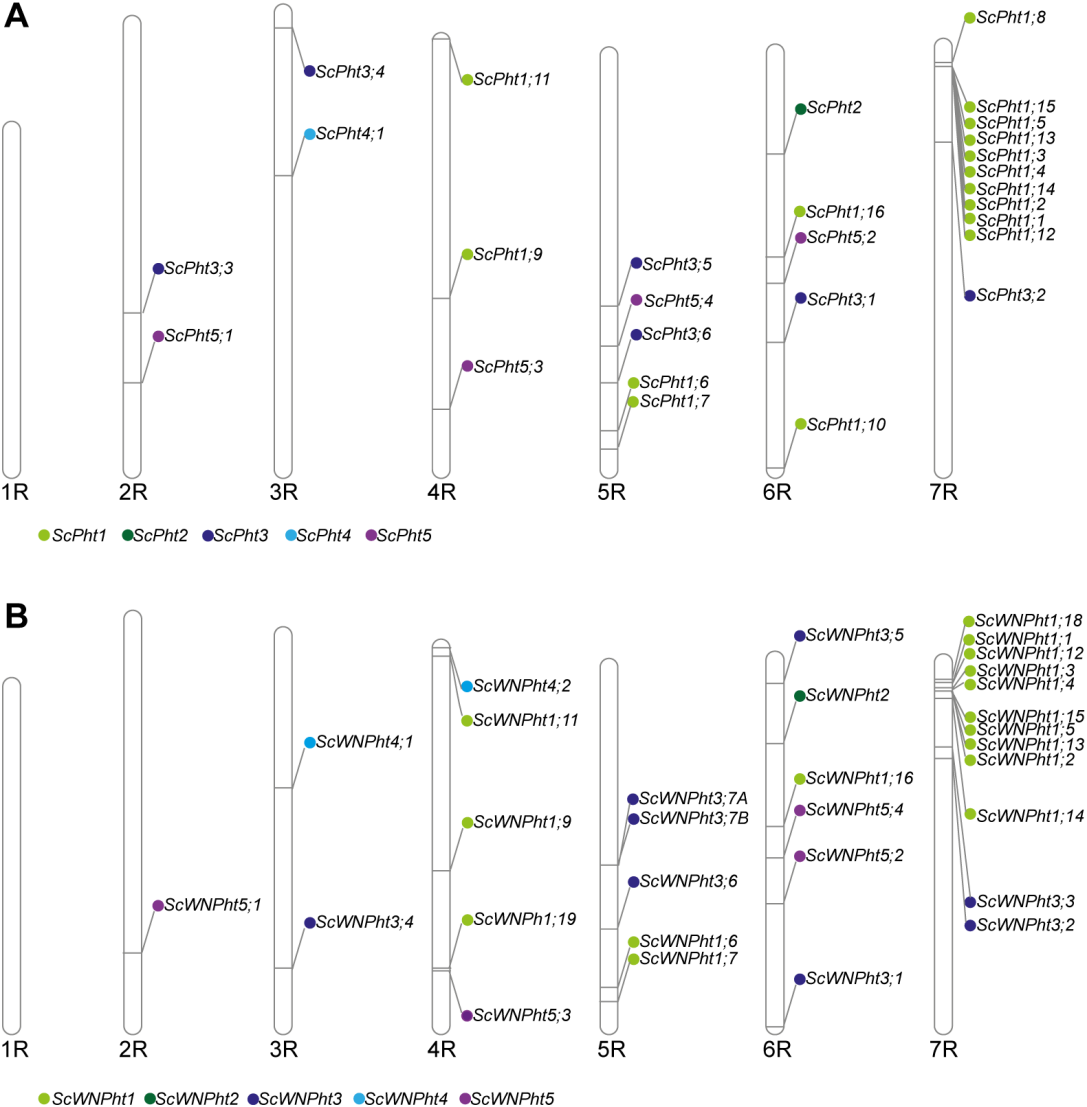
Distribution of *ScPht* genes on the rye Lo7 and Weining chromosomes. **(A)** Overview of the seven rye Lo7 chromosomes and the *ScPht* genes location. **(B)** Overview of the seven Weining Lo7 chromosomes and the *ScPht* genes location.

**Figure 2:**
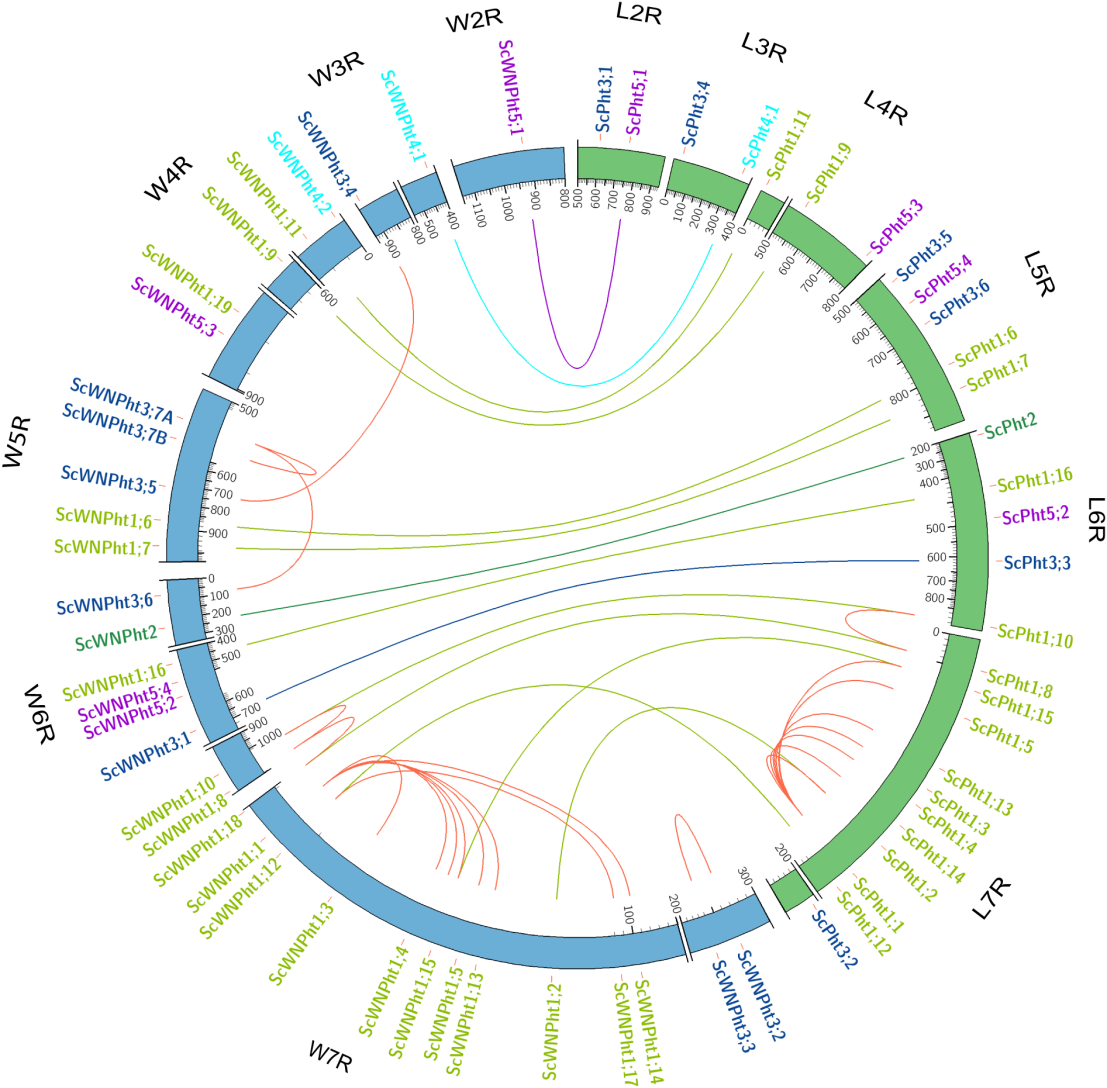
Distribution of *ScPht* genes on the rye chromosomes. Lo7 and Weining chromosomes are shown in green and blue, respectively. Only chromosome segments containing *Pht* genes are shown, at varying magnification, to ensure sufficient resolution. The scale is in kMb. Green, blue and purple lines indicate putative orthologs within each *Pht* family. Red lines indicate putatively duplicated genes.

We identified 29 and 35 putative *Pht* transporter genes in the rye Lo7 and Weining reference genomes, respectively (**Supplementary Tables S3**, **S4**). Both reference genomes possess one *Pht2*, two *Pht4* and four *Pht5* members. However, the Lo7 and Weining genomes differ in the number of members of the *Pht1* and *Pht3* families. The rye Lo7 genome contains 16 *Pht1* and 6 *Pht3* members, while the rye Weining genome holds 19 *Pht1* and 9 *Pht3* putative transporter genes (**Figures 1**, **2**). We used a phylogenetic tree-based approach to assign rye *Pht* gene names (*ScPht*) and infer orthology to Arabidopsis, rice, and between Lo7 and Weining *Pht* family members. We also took into account the sequence similarity – protein and nucleotide – to confirm orthology between Lo7 and Weining PHT family members (**Supplementary Tables S5**, **S6**, **Figure 2**). Both in the Lo7 and Weining genomes the *Pht* genes are distributed across all chromosomes, except for the chromosome 1R. Most *Pht* genes are located on chromosome 7R. However, a higher number of *Pht* genes was observed in Weining compared to Lo7 on chromosomes 4R, 6R and 7R. We identified several paralogs in the *Pht1* family in the Lo7 and Weining genomes (**Figure 2**). In Lo7, eight *Pht1* genes (*ScPht1;1*, *ScPht1;2*, *ScPht1;3*, *ScPht1;4*, *ScPht1;5*, *ScPht1;13*, *ScPht1;14*, and *ScPht1;15*), sharing > 95% nucleotide sequence similarity, cluster into a 1.93 Mb region in the chromosome 7R containing only two non-phosphate transporter coding genes. In this paralog group, several gene pairs are located within ~200 Kb from each other, indicating they arose from tandem duplication events (Houb, 2001; Freeling, 2009). Another Lo7 *Pht1* paralog pair – *ScPht1;8* and *ScPht1;10* – might have originated through segmental duplication since these highly similar genes (> 90% nucleotide sequence similarity) are located in different chromosomes. In the Weining genome, we detected a cluster of six *Pht1* genes (*ScWNPht1;2*, *ScWNPht 1;3*, *ScWNPht 1;4, ScWNPht 1;5*, *ScWNPht1;13*, and *ScWNPht1;15*) in chromosome 7R as a tandem duplication event. The paralog group *ScWNPht1;8*, *ScWNPht1;10* and *ScWNPht1;18* and the paralog pair *ScWNPht1;12*, *ScWNPht1;17* duplication nature could be considered as segmental duplication. Unlike Lo7, the Weining *Pht3* family underwent several duplication events. Two of these duplication events could be described as segmental duplications (*ScWNPht3;2/ScWNPht3;3* and *ScWNPht3;4/ScWNPht3;6*). Another paralog group composed of *ScWPht3;5*, *ScWNPht3;7A* and *ScWNPht3;7B* could have originated through both tandem and segmental duplication events.

### Synteny and collinearity analysis

Colinearity analysis between Lo7 and Weining genomes revealed no apparent large chromosomal translocation events. However, differences in chromosomal location of homologous genes between genomes were apparent across all chromosomes (**Supplementary Data Sheet 1**, **Figure S1A**). Interestingly, various Lo7 genes without assigned locations have Weining orthologs with defined chromosomal locations (**Supplementary Data Sheet 1**, **Figure S1B**). One of those genes is *ScPht4;2*, whose Weining ortholog (*ScWNPht4;2*) lies on chromosome 4R. Our results also showed that the *Pht*-bearing regions are syntenic between Lo7 and Weining (**Supplementary Data Sheet 1**, **Figure S1B**).

### Phylogenetic analysis of PHT transporter families

**Figure 3:**
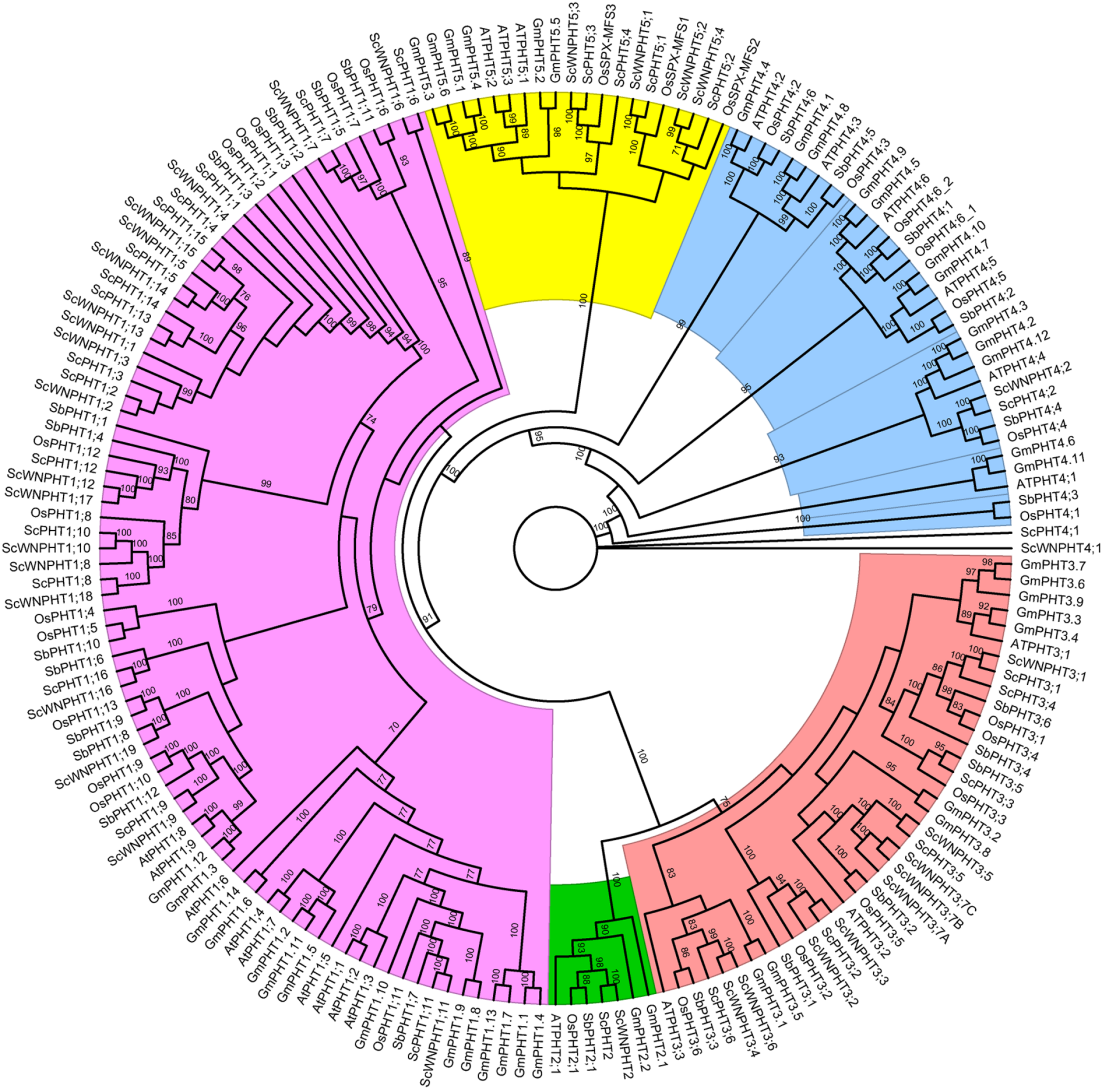
Phylogenetic relationships of PHT protein families from *Secale cereale* L. Lo7 and Weining, *Oryza sativa*, *Sorghum bicolor*, *Glycine max* and *Arabidopsis thaliana*. The protein tree was constructed using the maximum likelihood method. with the WAG+G4+F as the best-fitting substitution model. The ultrafast bootstrap and single branch test were inferred from 1000 replicates.

We constructed an unrooted phylogenetic protein tree using the Maximum likelihood method and containing a total of 184 PHT sequences from Arabidopsis (22 members), rice (30), sorghum (25), soybean (43), and both rye Lo7 (29) and Weining (35) to investigate the evolutionary relationships among PHT transporters. Our results showed that the members of the same PHT family from different plants cluster together, forming five clades (**Figure 3**). The largest clade - PHT1 - contains 83 members and a rye-specific subgroup contains 16 PHT1 members (eight for each rye genome). The PHT2 clade is the smallest and comprises seven members.

**Figure 4:**
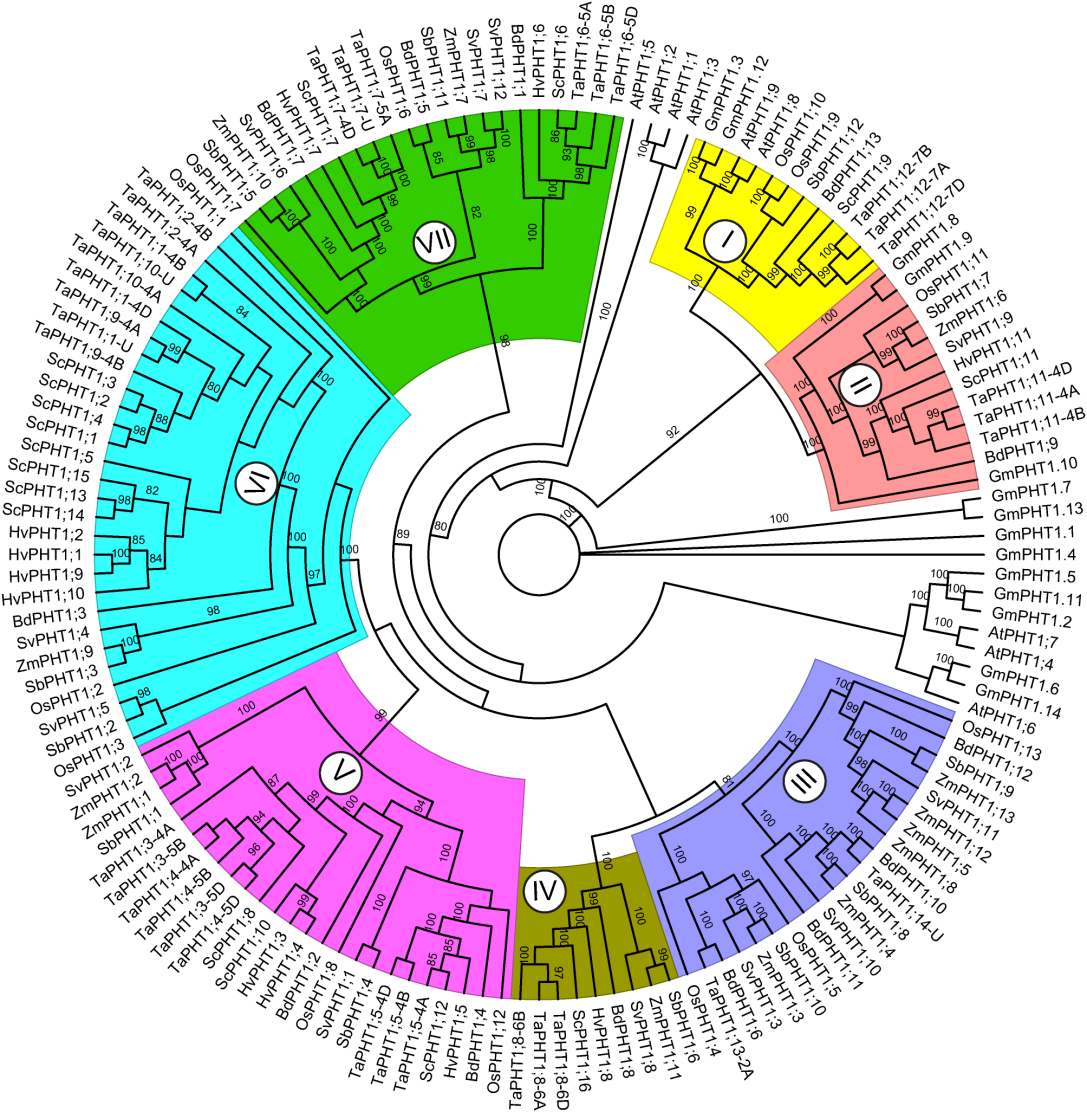
Phylogenetic relationships of PHT1 proteins from different monocot grass species. The phylogenetic protein tree contains PHT1 proteins from *Secale cereale* L., *Triticum aestivum*, *Hordeum vulgare*, *Oryza sativa*, *Sorghum bicolor*, *Zea mays*, *Setaria viridis*, *Brachypodium distachyon*, *Glycine max* and *Arabidopsis thaliana*. The protein tree was constructed using the maximum likelihood method with the JTT+R5 as the best-fitting substitution model. The ultrafast bootstrap and single branch test were inferred from 1000 replicates.

We further evaluated the PHT1 phylogenetic relationships among grasses to predict the potential function of rye PHT1 transporters (**Figure 4**). Our analysis showed that PHT1 transporters form seven clusters. Cluster I and II group PHT1 members from monocots and dicots, including a couple of ScPHT1s (one rye phosphate transporter in each clade). Cluster III does not hold any rye PHTs, while cluster VI includes the most rye phosphate transporters.

### Identification of the *cis*-acting regulatory elements

**Figure 5:**
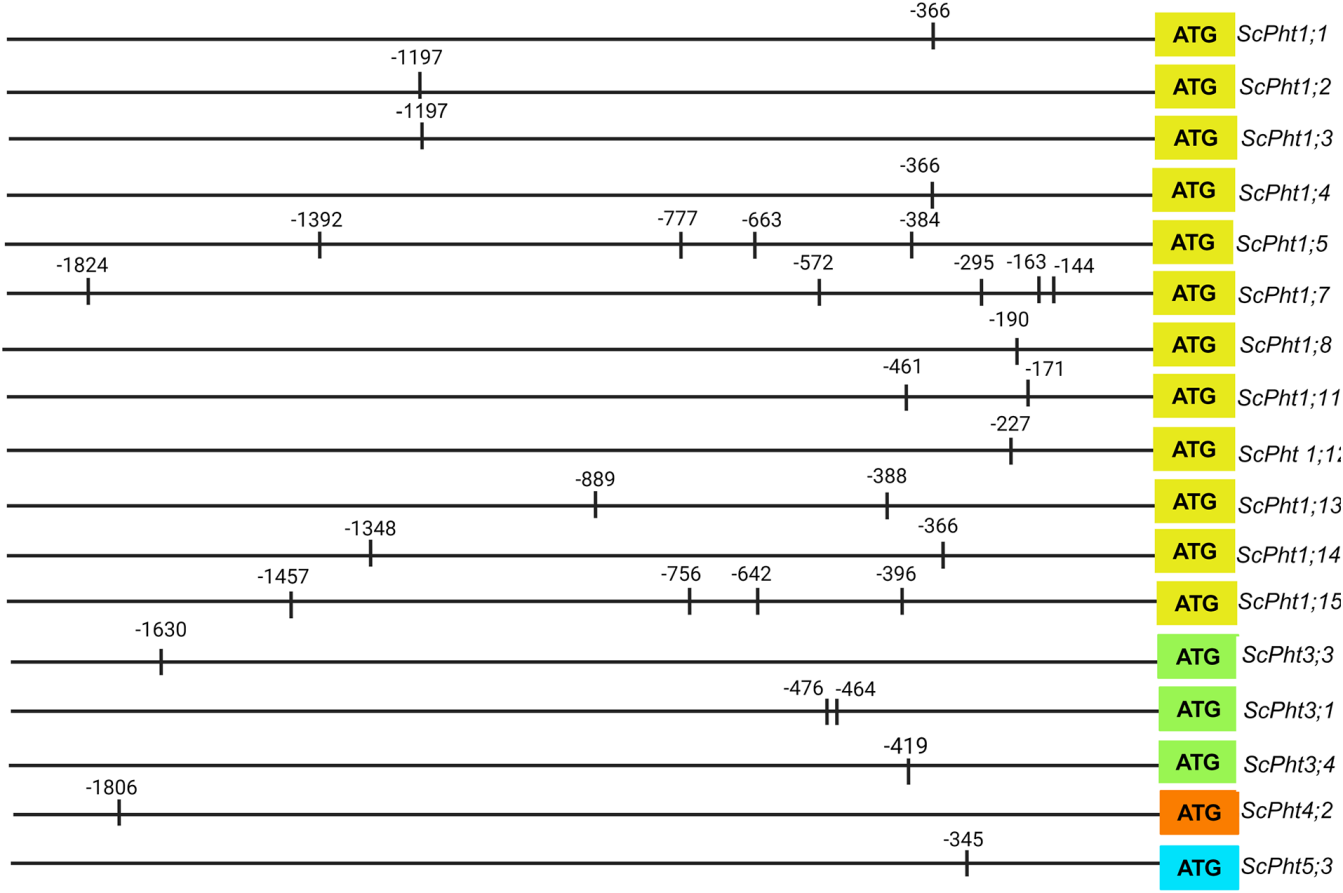
Location of P1BS *cis*-regulatory elements in the promoter region of *ScPht*s genes.

We identified at least one PHR1-binding sequence (P1BS) in the promoter region of 12 Lo7 and Weining *Pht1*s (**Figure 5**; **Supplementary Table S7**). The *ScPht1;5*, *ScPht1;7*, and *ScPht1;15* promoters – and their Weining orthologs – contain the highest number of P1BS *cis*-elements. Both the Lo7 and Weining *Pht2* do not hold any P1BS *cis*-element. In the *Pht3* family, the Lo7 and Weining *ScPht3;1*, *ScPht3;3*, and *ScPht3;4* contain two, one, and one P1BS *cis*-element, respectively. We found the phosphate deficiency responsive regulatory element in only one member of the Lo7 *ScPht4* and *ScPht5* families (*ScPht4;2* and *ScPht5;3*). Conversely, no Weining *Pht4* and *Pht5* family members carry any P1BS-*cis* elements.

### Expression profiles of rye phosphate transporters in leaves and roots

Quantitative RT-PCR was used to analyze the responses of *ScPht* genes to Pi deficiency at 14 and 21 days of hydroponic cultivation (corresponding to 20 and 27 days after germination) in the inbred line Lo7 leaves and roots. We selected *ScPht1;6, ScPht1;7*, and *ScPht1;11* for primer design because they contain P1BS *cis*-elements and exhibit lower nucleotide sequence similarity to each other: *ScPht1;6* shares 77.8% similarity with *ScPht1;7* and 69.13% with *ScPht1;11* (**Supplementary Table S8**). We also selected *ScPht3;1*, *ScPht3;3*, and *ScPht3;4* which display higher sequence similarities (up to 84.69%), and *ScPht5;1* and *ScPht5;3*, which share 40.61% similarity. We designed primers for *ScPht1;6*, *ScPht1;7*, *ScPht1;11*, *ScPht2*, *ScPht3;1*, *ScPht3;3*, *ScPht3;4*, and *ScPht5;3* (**Supplementary Table S9**), but only the *ScPht1;6*, *ScPht2*, and *ScPht3;3* primers demonstrated high efficiency experimentally.

**Figure 6:**
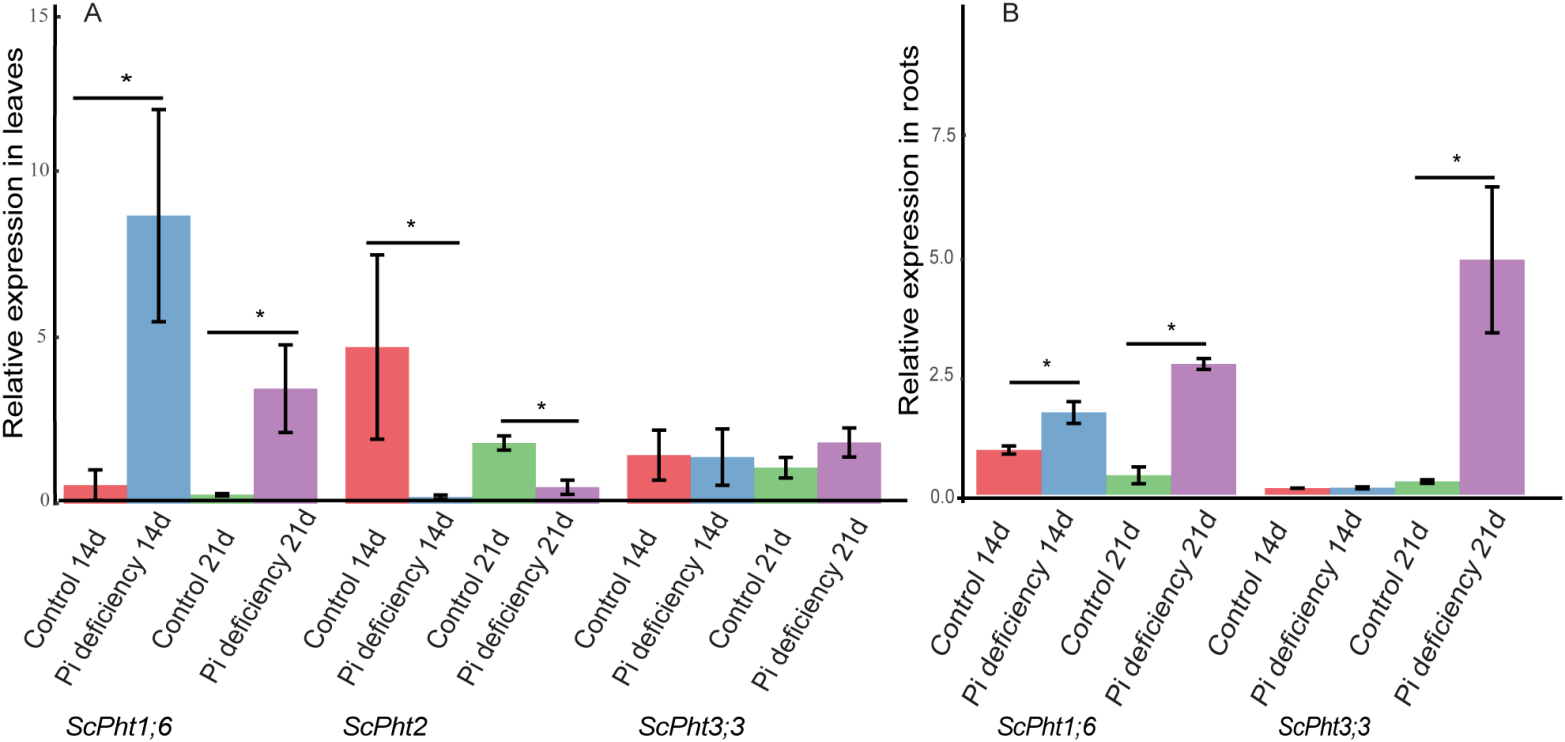
Relative expression levels of phosphate transporter genes assessed by qRT-PCR in leaf **(A)** and root **(B)** tissue under two treatment conditions: phosphate deficiency (Pi deficiency) and phosphorus sufficiency (control), at two-time points (14 days and 21 days). Each bar plot represents the mean 2^-ΔΔCt values obtained from three independent biological replicates, with error bars indicating the standard error of the mean (SEM). Statistical significance between control and treatment conditions was determined using the Kruskall test (*p < 0.05).

In leaf tissue, *ScPht1;6*, *ScPht2*, and *ScPht3;3* showed different expression patterns (**Figure 6A**). *ScPht1;6* was significantly upregulated under Pi deficiency at 14 and 21 days, while *ScPht2* was significantly downregulated at both time points. *ScPht3;3* showed low expression with no significant differences between treatments.

In root tissue (**Figure 6B**), only *ScPht1;6* and *ScPht3;3* were differentially expressed, while *ScPht2* was not detected. *ScPht1;6* was significantly upregulated under Pi deficiency at 14 and 21 days. *ScPht3;3* was significantly upregulated at 21 days, but at 14 days, its expression was low with no significant differences.

### Low coverage resequencing (DArTreseq): phylogenetic relationships among 94 diverse rye accessions and sequence diversity of *ScPht* transporters

**Figure 7:**
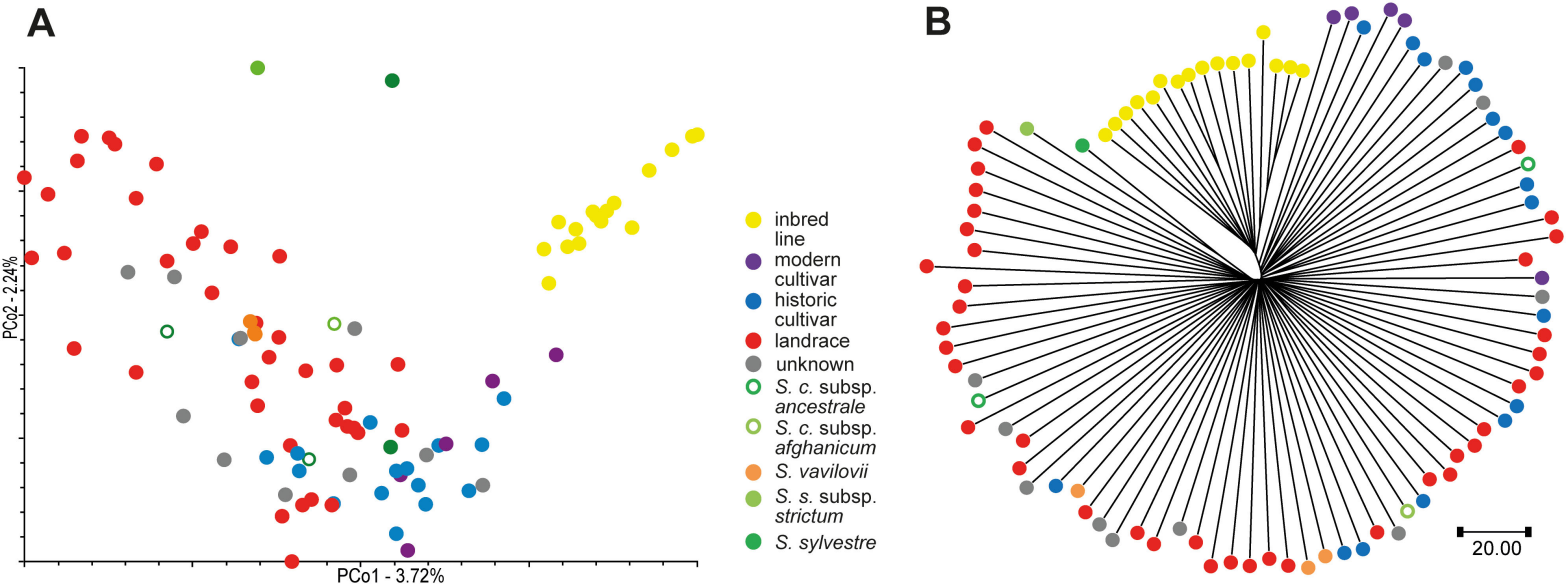
Genetic diversity of 94 rye accessions analyzed by DArTreseq. **(A)** PCoA plot based on 190430 genome-wide SNPs. **(B)** NJ tree based on 190430 genome-wide SNPs.

Initially, ca. 2,5 Mio. SNPs differentiating the 94 rye accessions included in the study were identified as a result of DArTreseq genotyping. After quality filtering 190–430 SNPs remained and were used for PCoA and NJ analyses. A high diversity of the collection was revealed (**Figures 7A, B**). Rye inbred lines separated from the remaining accessions, while landraces overlapped partially with cultivars, but displayed much higher diversity.

**Figure 8:**
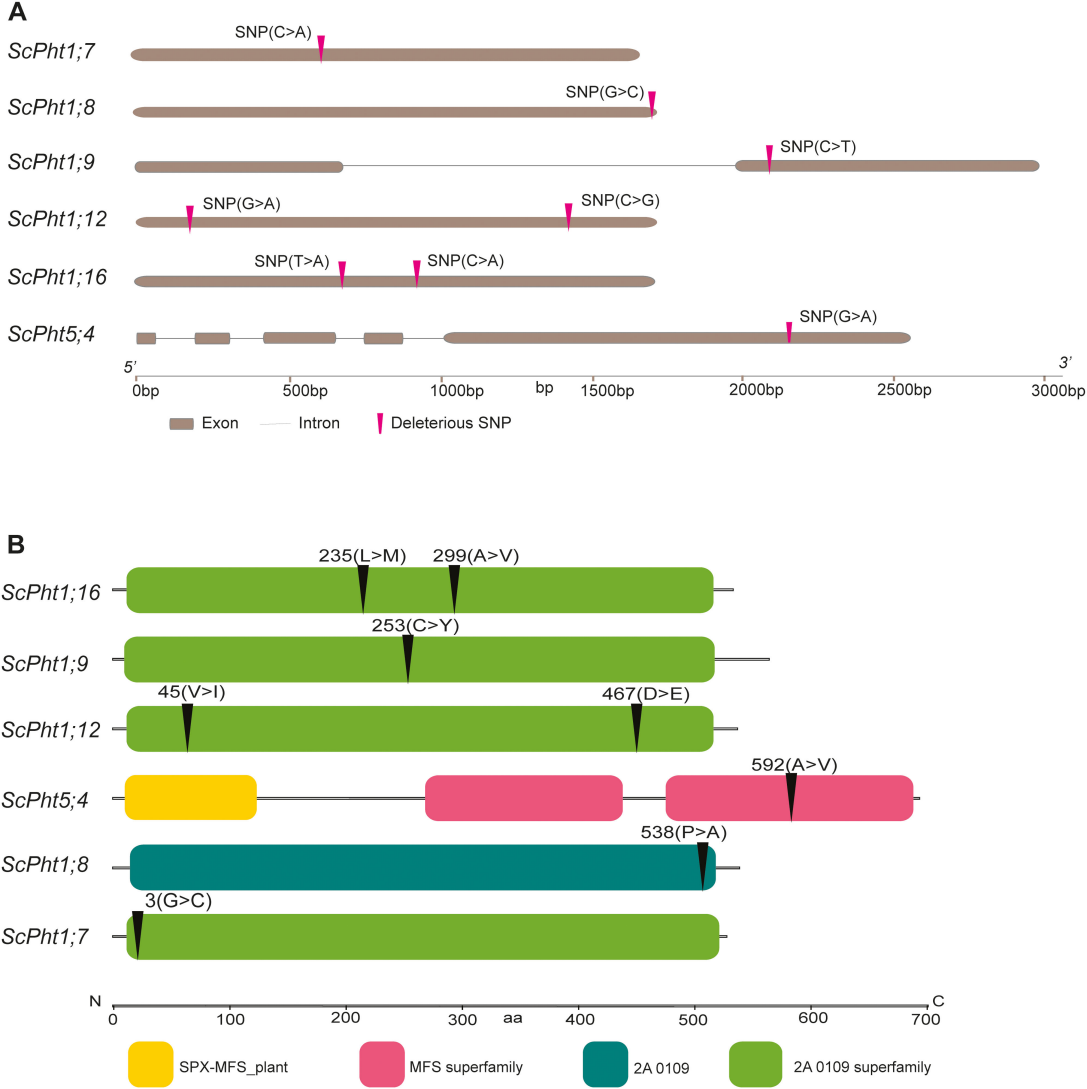
Diagram of the nonsynonymous deleterious SNP location within the *ScPht* members. **(A)** Location of deleterious SNP within the coding regions of *ScPht* genes. **(B)** Amino acid changes due to deleterious SNPs in the protein sequences of PHT transporters.

In total 820 polymorphic sites were observed within the putative 29 *ScPht* genes identified in the Lo7 genome. All identified polymorphisms were biallelic SNPs, with exception of two loci, where two alternative alleles were discovered. Transitions (G to A, C to T, A to G and T to C changes) constituted almost 54% of the discovered polymorphisms. The cumulative length of the analyzed genes and their putative regulatory regions was 185–414 bp, which corresponds to overall SNP density 1 SNP every 226 bp. The highest SNP density was observed in introns (one SNP per 128 bp), followed by CDS (one SNP per 187 bp) and UTRs (one SNP per 297 bp on average) (**Supplementary Table S10**). None of the UTR SNPs were located within the P1BS *cis*-regulatory elements. No polymorphisms were identified in the genes *ScPht1;2*, *ScPht1;3*, *ScPht1;4* and *ScPht1;14*. Within the putative CDSs no SNP were identified in eight *ScPht1* genes– *ScPht1;1* - *ScPht1;5* and *ScPht1;13* - *ScPht1;15.* The number of polymorphic sites within *ScPht* genes per accession ranged from 51 (in inbred line L318) to 201 in the landrace NSL308 from Turkey, 131 on average. The number of accessions with a given variant ranged from 1 to 70, on average a variant occurred in 15 accessions. In total 11 private variants (occurring only in a single accession from the set) were identified. Six of the private variants occurred in wild/weedy accessions, four in landraces, and one in a historic cultivar. The private variants were identified in ten genes: *ScPht1;1*, *ScPht1;5*, Sc*Pht1;9*, *ScPht1;11*, *ScPht3;1 ScPht3;3*, *ScPht3;5*, *ScPht4;1*, *ScPht5;1*, and *ScPht5;2*. The majority of private variants were located in UTR regions, only the private variants in *ScPht3;3* and *ScPht4;1* were located in introns. Overall 48 SNPs located in the CDS of *ScPht* genes resulted in amino acid changes. In total 12 SNPs putatively deleterious missense variants were predicted by SIFT4G analysis in seven *ScPht* genes (**Supplementary Table S11**). Of these, 8 are nonsynonymous changes including two scored as high confidence. The deleterious variants were identified in the genes: *ScPht1;7*, *ScPht 1;12*, *ScPht 1;16* (two variants in each) and in genes: *ScPht1;8, ScPht1;9, ScPht1;10, ScPht3;5, ScPht5;3* and *ScPht5;4* (one variant in each). The number of accessions with a given deleterious variant ranged from three to 26. The location of nonsynonymous, deleterious SNPs and corresponding amino acid changes is shown in **Figure 8**.

## Discussion

The distribution of phosphate to the various plant tissues and cellular compartments depends on PHT transporters. In our study, we surveyed rye Lo7 and Weining reference genomes to identify members of the *Pht* transporter families. The number of *Pht* genes in each rye reference genome – 29 and 35 in Lo7 and Weining, respectively – differs, particularly among the *Pht1* and *Pht3* family members. These discrepancies in the *Pht* gene numbers between reference genomes could be due to genomic diversity (several gene duplication events) or *de novo* genome sequencing approaches.

Genetic diversity arises from small (e.g. single nucleotide polymorphism, insertion/deletions) and large-scale (more than 50 bp and up to several Mb) polymorphisms (Saxena et al., 2014). The large-scale polymorphisms, also known as structural variations (SVs) include copy number variation (CNV), insertions, deletions, duplications, inversions, intra and interchromosomal translocation (Ho et al., 2020).

Several studies have uncovered structural variation – specifically the presence or absence of loci – conferring advantageous agronomical and adaptive traits (Yuan et al., 2021; Zanini et al., 2022), such as the previously mentioned the *Pup1* locus, providing advantages under low-P environments (Heuer et al., 2009; Gamuyao et al., 2012). These examples point out the risk of overlooking genetic variants associated with adaptive traits when relying on a single reference genome.

In rye, three-dimensional conformation capture sequencing (Hi-C) revealed large inversion between rye Lo7 and Lo255 inbred lines (Rabanus-Wallace et al., 2021) and between Lo7 and rye wild relative *Secale strictum* (Rabanus-Wallace et al., 2021). However, precise data on the extent of structural variation regarding the presence/absence variation of gene loci are not available yet for rye. The variation in the *Pht* gene number between rye Lo7 and Weining reference genomes might reflect the adaptation to their local environments.

The Lo7 and Weining genomes were sequenced using Illumina short reads and PacBio, respectively (Li et al., 2021; Rabanus-Wallace et al., 2021). Although Illumina sequencing platforms have been the workhorse of several *de novo* genome sequencing projects, the short-length read imposes challenges when assembling large and complex plant genomes (Kong et al., 2023). A large content of repetitive sequences (in rye represent around 90% of the genome) (Schatz et al., 2012; Bauer et al., 2017) and the existence of large gene families add a layer of difficulty to genome assembly (Schatz et al., 2012). As shown in our study, many members of the *Pht1* family – in both Lo7 and Weining genomes – share a high nucleotide sequence similarity (> 90%) among them. Thus, the short-sequencing-based Lo7 genome nature might have underestimated the numbers of *Pht1* and *Pht3* family members.

We also observed variation in the number of members from each *Pht* family across plant species. For instance, both rye reference genomes – 16 *Pht1* in Lo7 and 19 *Pht1* in Weining – hold a larger number of *Pht1* genes compared to Arabidopsis (9 *Pht1*), rice (13 *Pht1*), barley (11), maize (13), brachypodium (13), *Setaria* (12), and sorghum (12 *Pht1*). The expansion of gene families is often attributed to fragment and tandem duplication as well as transposable replication and whole genome duplication (WGD) events (Fang et al., 2022). The rye Weining genome contains a higher number of proximal and tandem duplication genes compared to other grasses - barley, diploid wheat, *Aegilops tauschii*, brachypodium, and rice (Li et al., 2021). Similarly, the transposed duplicated gene number in rye Weining is considerably larger than in diploid wheat and *Aegilops tauschii.* Some rye Weining genes involved in starch biosynthesis had undergone transposed (*ScSSIV*, *ScDPEI*, *ScSuSy1*, *ScSuSy1*, and *ScUDPaseI*), tandem (*ScPHO2*), proximal (*ScAGP-L2-p*, and *ScSBE1*), and dispersal (*SSIIIa*) duplication events (Li et al., 2021).

Through our phylogenetic analysis (**Figure 4**), we could hypothesize about the role of rye PHT in P homeostasis, since several PHT1 transporters have been experimentally validated. For instance, cluster VI contains various wheat (TaPHT1.1, TaPHT1.2, TaPHT1.9, and TaPHT1.10) and barley (HvPHT1.1) transporters exhibiting Pi-transport activity, and increased expression in roots under Pi deficiency (Rae et al., 2003; Teng et al., 2017). The PHT1 phylogenetic protein tree also revealed interesting relationships between rye, maize, and rice phosphate transporters. For example, the rye ScPHT1;6 closely relates to HvPHT1.6. The barley PHT1;6 and rice PHT1;7 transporters have been suggested to remobilize stored Pi from old to young leaves (Rae et al., 2003; Dai et al., 2022). Moreover, the putative rye ScPHT1;9 transporter groups with the functionally redundant rice OsPHT1;9 and OsPHT1;10 – both transporters are involved in Pi-uptake (Wang et al., 2014a). The cluster II groups ScPHT1;11 with OsPHT1;11 and ZmPHT1;6 – both transporters are known to be involved in mycorrhiza-dependent Pi-uptake (Zhu et al., 2012; Willmann et al., 2013).

To determine what rye *Pht* genes are Pi-deficient responsive and potentially involved in P-homeostasis, we evaluated the *ScPht* gene expression in leaves and roots under Pi-deficient conditions. Genes were selected for qRT-PCR analysis based on the presence of P1BS *cis*-elements, but the high sequence similarity within *ScPht* gene families limited gene-specific primer design. Despite lacking a P1BS *cis*-element, *ScPht2* was included due to functional evidence from its orthologs in Arabidopsis, wheat, and sorghum, emphasizing its role in Pi transport (Versaw and Harrison, 2002; Guo et al., 2013; Wang et al., 2019).

Under Pi-starvation stress, the expression of *Pht1* genes is significantly upregulated to maximize the root Pi absorption and facilitate Pi remobilization within the plant (Smith et al., 2003; Raghothama and Karthikeyan, 2005). Our study found significant upregulation of *ScPht1;6* in roots under Pi deficiency at 14 and 21 days (**Figure 6B**). *ScPht1;6* also exhibited a significant upregulation in Pi-deficient leaves compared to control samples at 14 and 21 days, with the highest expression levels observed at 14 days of Pi deficiency (**Figure 6A**). Our phylogenetic analysis identified *ScPht1;6* as an ortholog of *TaPht1;6-5A* in wheat (**Figure 4**). *TaPht1;6-5A* exhibited differential organ-specific expression, enhancing Pi acquisition and accumulation in all organs of a Pi-efficient wheat cultivar under Pi withdrawal (Aziz et al., 2014). Similarly, *OsPht1;6*, the rice ortholog of *ScPht1;6* (**Figure 4**), exhibited significant upregulation after 14 days of low P conditions (Anandan et al., 2021). In barley, *HvPht1;6*, an ortholog of *ScPht1;6* (**Figure 4**) showed significantly elevated transcript levels at 17 days under severe Pi deficiency in shoots and roots (Huang et al., 2008). This similarity in expression patterns under Pi deficiency suggests the involvement of *ScPht1;6* in P homeostasis.

We also examined the expression of *ScPht2*. This gene was not expressed in roots but displayed significant downregulation in leaves under Pi deficiency. *ScPht2* expression remained low with no significant variations over time in P-deficient conditions. These results suggest that *ScPht2* may not play a direct role in Pi uptake during Pi stress but could be essential for maintaining Pi homeostasis under normal conditions. On the contrary, in wheat and rice the expression level of *TaPht2;1* and *OsPht2;1*, respectively, was enhanced in leaves under Pi deficiency (Guo et al., 2013; Liu et al., 2020). Likewise, the expression of two *GmPht2* genes was induced by low-Pi stress in soybean leaves (Wei et al., 2023). Overall, these findings underscore the diverse transcriptional regulation of *Pht2* genes in Pi deficiency across different plant species.

The plant gene family *Pht3* is known to localize in the mitochondrial inner membrane (Jia et al., 2015). In our study, *ScPht3;3* expression levels in leaves showed no significant differences between treatments at both time points, indicating it is not dependent on Pi availability (**Figure 6A**). Conversely, *ScPht3;3* expression levels in roots displayed a significant upregulation at 21 days of Pi deficiency, indicating a response to prolonged Pi deprivation in the root system. In sorghum, *SbPht3;6* was upregulated more than 2-fold in the leaves and downregulated in roots after 14 days of Pi starvation (Wang et al., 2019). In pepper, differential expression of *CaPht3* was observed in both roots and leaves during P deficiency (Ahmad et al., 2021). The contrasting expression patterns of the *SbPht3;6* and Ca*Pht3* genes highlight species-specific regulatory mechanisms of *Pht3* transporters under Pi stress.

Our qRT-PCR data indicate that the analyzed *ScPht* genes are Pi-deficiency responsive. To better characterize functions of the identified *ScPht* genes in Pi uptake and translocation, further research is needed, involving spatiotemporal expression patterns and functional analyses.

We have analyzed the genetic diversity of the identified *ScPht* genes in a collection of 94 rye accessions with various improvement status and geographic origins using low coverage resequencing (DArTreseq). Accessions originating from different cultivation environments were included in the set, for example, landraces/historical cultivars from areas such as Brazil, Norway, Finland or Sweden, where the very high P-retention potential of the soil results in a low availability of P for plants (Kochian, 2012), as well as modern varieties bred for cultivation in Central Europe (Germany, Poland), where P supply is not a limiting factor.

Neighbor Joining and Principal Coordinates analyses revealed a large diversity of the collection. The obtained picture of genetic diversity patterns is in good agreement with results of previous studies on rye genetic diversity (Bolibok-Brągoszewska et al., 2014; Monteiro et al., 2016; Hawliczek et al., 2020, 2023) which indicated genetic distinctness of rye inbred lines, broad diversity of genetic resources and narrower diversity of historical and modern rye varieties. Therefore it can be assumed that the collection is representative and covers a broad spectrum of rye genetic diversity. Nevertheless, the average SNP density observed in this study across the *ScPht* genes (1 SNP every 226 bp) was much lower than reported earlier for rye by one SNP or InDel every 12 bp (Hawliczek et al., 2020), 1 SNP/52 bp (Li et al., 2011), and 1 SNP/58 bp (Varshney et al., 2007). Very likely the technical limitation of the applied approach of probing the gene diversity used in the present study (low coverage resequencing in a large genome species) has contributed to the lower SNP density to some extent (Bilton et al., 2018), however, the observed differences in SNP density could also reflect differences in mutation rates across the genomic regions analyzed in the studies mentioned above. It has been observed that mutation rates vary in plant genomes, with differences occurring across genotypes, genome locations, gene functions, etc. (Quiroz et al., 2023) and it is assumed that the coding sequences of developmental genes are strongly conserved, whereas genes with roles in defense evolve more rapidly (Man et al., 2023). While *phosphate transporter* genes are the sole focus of the present study, the earlier analyses involved: six genes related mostly to biotic stress resistance and seed quality (Hawliczek et al., 2020), 12 genes with putative roles in frost tolerance (Li et al., 2011), 14 ESTs with various putative functions (Varshney et al., 2007).

Furthermore, SNP density varied markedly within each *ScPht* family: for example, the *ScPht1* family comprised genes with SNP density ranging from 1 SNP/84 bp to 1 SNP/795 bp, as well as three genes where no polymorphism was detected. These findings might reflect the evolutionary history of these gene families, involving recurrent gene duplication and accumulation of genetic diversity in some copies by positive natural selection or other mechanisms (Long, 2001; Ohta, 2013). Moreover, different mutation rates can be a consequence of such factors as chromosomal location, gene orientation or transcriptional activity (Lynch et al., 2016).

In total, 11 private variants were identified within *ScPht* genes. The majority of those private variants occurred in wild accessions or landraces, which further confirmed the value of crop wild relatives and landraces as a source of potentially useful variation in rye and other crops (Dwivedi et al., 2016; Zhang et al., 2017; Hawliczek et al., 2020). It was shown in the past that landraces are an important source of genes enhancing nutrient uptake and utilization (Dwivedi et al., 2016). Furthermore, 12 of the variants were flagged to be potentially deleterious to gene function, with three variants receiving high confidence SIFT scores. Interestingly, of the 12 variants, four are synonymous changes. These are likely mis-predictions as SIFT evaluates the effect of amino acid changes. Such errors can occur due to low-sequence diversity of evaluated proteins or false positive errors when using the SIFT threshold of 0.05, that aims to increase sensitivity for deleterious changes while allowing for false positive signals (Gupta et al., 2020; Luppino et al., 2023). Use of additional prediction tools may be useful in balancing false-negative and false-positive errors. Interestingly, potentially deleterious synonymous changes are being reported in the human exome, and the phenotypic effect of such variants has been experimentally tested in yeast (Shen et al., 2022; Bilton et al., 2018). Thus, in addition to the handful of high confidence nonsynonymous deleterious changes reported here, many more functionally important variants may be discovered as computational tools improve. Therefore, it can be argued that, despite certain shortcomings (low coverage, disregarding intra-accession diversity), the adopted approach of detecting sequence diversity (DArTreseq) allows for a quick and relatively cost-effective insight into the diversity of all annotated genes, and produces data which permit for relative comparison of polymorphism levels across genes/gene families of interest and quick identification and prioritization of candidates for further functional studies.

Rye is a close relative of wheat and over decades rye has been effectively used in wheat breeding as a source of variation, especially to enhance tolerance of various biotic and abiotic stresses. As a result, the proportion of wheat varieties carrying rye chromatin exceeds 30% in some countries (Crespo-Herrera et al., 2017). Wheat is a cereal of global economic importance, grown worldwide, also in the areas, where Pi-deficiency is a considerable constraint to agricultural production, such as India. Various efforts to improve wheat’s P-deficiency tolerance are ongoing (Soumya et al., 2021; Dharmateja et al., 2022). Rye has been shown to have a higher P-deficiency tolerance than wheat (Pandey and Singh, 2005; Pandey, 2006). Therefore, the description of the role of the *ScPht* genes and their different allelic variants in Pi uptake and distribution and the understanding of their contribution to plants’ Pi-deficiency response would be of relevance also for wheat breeding – for the development of more Pi- efficient and P-deficiency tolerant cultivars.

## Conclusions

Rye genome contains putative phosphate transporter genes from all known *Pht* families. Phylogenetically, putative rye transporters exhibit a close relationship to previously functionally characterized PHT proteins from other grasses. The two available rye reference genomes differ in the number of *Pht1* and *Pht3* genes and the polymorphism level varies markedly among the identified *ScPht* genes within the rye diversity panel, suggesting multiple layers of adaptation to local environments existing in rye. *ScPht1;6, ScPht2* and *ScPht3;3* genes are responsive to Pi-deficiency, which might suggest their involvement in Pi homeostasis. DArTreseq genotyping permits for a quick and cost-effective assessment of polymorphism levels across genes/gene families and supports identification and prioritization of candidates for further studies.

Our report is the first step toward elucidating the mechanisms of rye’s response to Pi deficiency. Collectively our findings provide the foundation for selecting most promising candidates for further functional characterization.

## Data Availability

FASTQ files related to DArTreseq analysis of 94 rye accessions generated and analyzed in this study are available in NCBI BioProject PRJNA1091674.
