# Supplementary material for: Phosphate transporter gene families in rye (Secale cereale L.) – genome-wide identification, characterization and sequence diversity assessment via DArTreseq
Source: Front Plant Sci. 2025 Jun 16;16:1529358. doi: 10.3389/fpls.2025.1529358 (PMC12206809; doi:10.3389/fpls.2025.1529358)

# Supplementary File 2

# Supplementary Figures

**Figure S1. A.** Collinearity analysis between Lo7 and Weining genomes. The 1R to 7R labels represent Lo7 chromosomes, while GWHASIY00000001 to GWHASIY00000007 represent the Weining chromosomes. The color lines represent the collinearity blocks between Lo7 and Weining chromosomes**. B.** Collinearity analysis between Lo7 genes without assigned locations and the Weining genomes. The 0R label represents the Lo7 chromosome unknown (ChrUn). The Weining chromosomes are labeled as GWHASIY00000001 to GWHASIY00000007. The lines connecting Lo7 and Weining chromosomes represent collinear blocks.


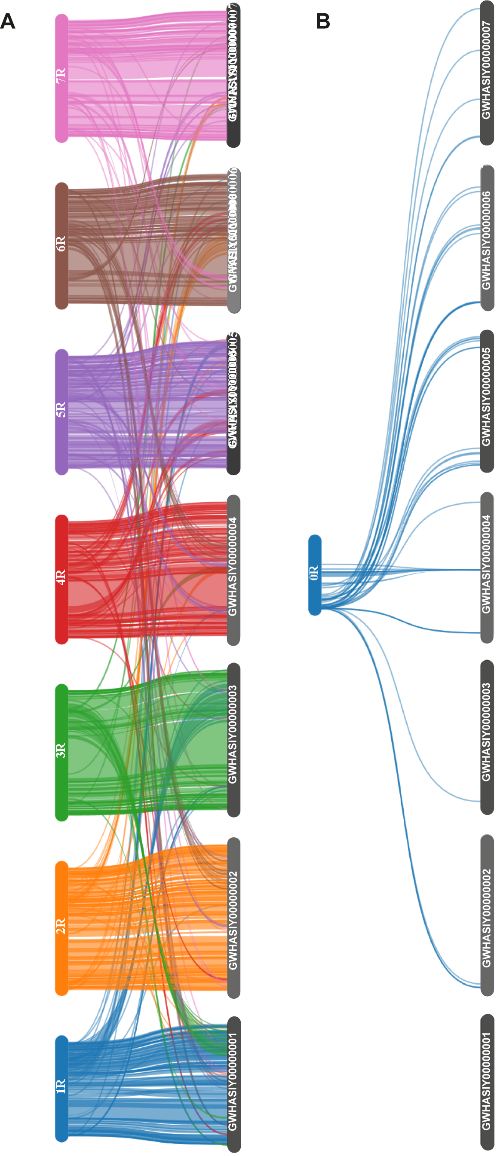


**Figure S2.** Syntenic relationships in the regions of rye genomes bearing the *ScPht* genes. Lo7 and Weining chromosomes are shown in green and blue, respectively. Only chromosome segments containing *Pht* genes are shown, at varying magnification, to ensure sufficient resolution. The scale is in kMb. Green, blue and purple lines indicate putative orthologs within each *Pht* family. Red lines indicate putatively duplicated genes. Grey lines connect positions of homologous genes from other families, identified by TBtools.


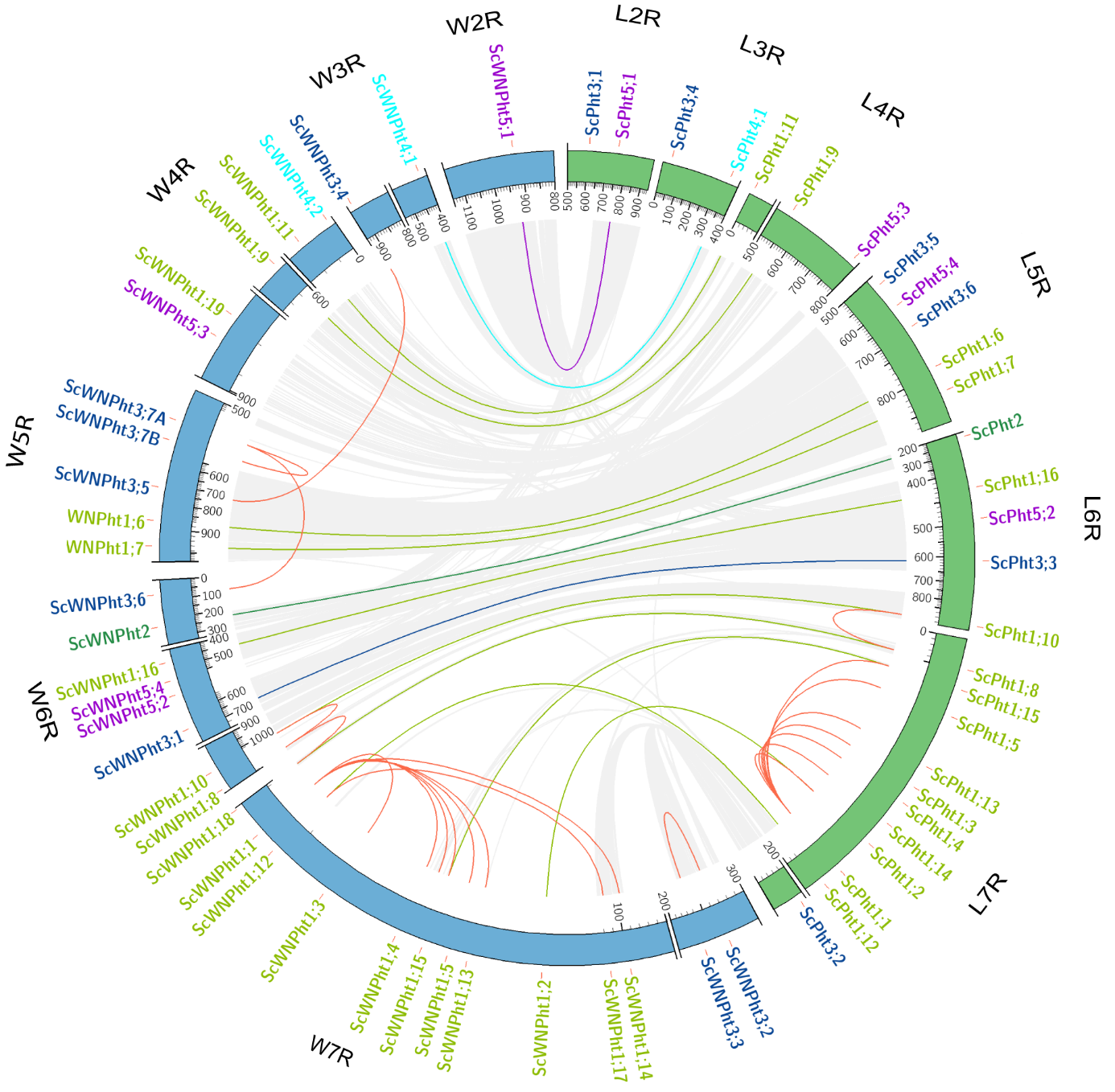

Supplement: Supplementary file 1 [file DataSheet1.docx]
